# Colorectal adenomas and diabetes: implications for disease prevention

**DOI:** 10.1111/codi.12895

**Published:** 2015-06-22

**Authors:** R. J. C. Steele, A. S. Anderson, M. Macleod, A. M. Craigie, S. Caswell, J. Belch, S. Treweek, on behalf of the BeWEL team

**Affiliations:** ^1^ Centre for Research into Cancer Prevention and ScreeningMedical Research InstituteNinewells Hospital and Medical SchoolUniversity of DundeeDundeeUK; ^2^ Vascular Diseases Research UnitMedical Research InstituteNinewells Hospital and Medical SchoolUniversity of DundeeDundeeUK; ^3^ Health Services Research UnitUniversity of AberdeenAberdeenUK

**Keywords:** Adenoma, screening, diabetes, obesity, cardiovascular

## Abstract

**Aim:**

This study assessed the baseline type II diabetes mellitus (T2DM) risk status among overweight patients with screen‐detected colorectal adenomas and explored the implications of the findings for preventative practice.

**Method:**

Participants aged between 50 and 74 years (73% of whom were men) were recruited from four Scottish health boards and assessed for diabetes risk. Participants were categorized as at ‘high’ diabetes risk if glycated haemoglobin (HbA1c) was between 6.0 and 6.4% or fasting plasma glucose (FPG) was between 5.5 and 6.9 mmol/l and as potentially *undiagnosed* T2DM when HbA1c ≥ 6.5% or FPG ≥ 7 mmol/l. Secondary outcome measures included anthropometric measurements, blood pressure and the plasma lipid profile. The tests were repeated at 12 months and diabetes risk categories were reassessed following intervention procedures.

**Results:**

Forty‐seven (14.3%) of the 329 participants had a preexisting diagnosis of T2DM. Of the remainder with complete biochemistry results (*n *=* *250), 19 (7.6%) were classified as having potentially *undiagnosed* T2DM and 125 (50.0%) as being at *high risk* of developing diabetes. More than a quarter of participants in all categories had raised waist circumference, hypertension and plasma lipids, indicative of raised cardiovascular risk. At 12 months' follow‐up, the diabetes risk category diminished in 20% of the intervention group *vs* 11% in the controls [OR 2.26 (95% CI 1.03–4.96)].

**Conclusion:**

Our results suggest that a diagnosis of adenoma in overweight patients provides a health service opportunity for diabetes assessment, prevention and management in a high‐risk population at a potentially teachable moment.

**What does this paper add to the literature?**

Increasing identification of patients at high risk of type 2 diabetes mellitus within the colorectal screening setting offers an unexplored opportunity for screening and lifestyle modification to reduce metabolic complications and related comorbidity in adults (particularly men) aged 50–74 years.

## Introduction

Colorectal cancer (CRC) is the third most common cancer and cause of cancer death in the UK 1. Most cases occur in people aged over 50 years, often coexisting with other chronic conditions including obesity, type 2 diabetes (T2DM) and cardiovascular disease (CVD). These diseases share common risk factors related to cardiometabolic abnormalities, including high levels of abdominal fat, abnormal blood lipid profiles, markers of insulin resistance, increased production of insulin, endothelial growth factors and adipocytokines 2, 3. Meta‐analyses have demonstrated a consistent association between obesity and CRC 4, 5 (notably in men) and with colorectal adenomas in men and women 6, 7. Furthermore, a number of papers have highlighted an association between metabolic syndrome and increased risk of CRC 8, 9, 10. A recent systematic review and meta‐analysis has reported a hazard radio (HR) of 1.26 (1.14–1.40) for the incidence of CRC in patients with diabetes 11. Whilst these findings highlight an important epidemiological association, the implications have not yet been considered in cancer prevention terms.

The Scottish government strategy to decrease the total CRC burden focuses on early disease detection and the national CRC screening programme offers biennial faecal occult blood testing (FOBT) to those aged 50–74 years 12. CRC screening detects colorectal adenomas which can be removed by endoscopic procedures. Whilst this reduces the risk of subsequent cancer, underlying modifiable risk factors (e.g. excess body weight) for chronic diseases including cancer, CVD and T2DM remain.

In 2011 there were 217 514 people in Scotland with T2DM, but there are also thought to be a further 50 000 undiagnosed and a higher number with a raised risk for developing the condition 13. There is no national diabetes screening programme, but practical and inexpensive routes to prevention and early detection are clearly highly desirable to delay both complications and the development of associated conditions such as CRC.

The aim of the current study is to assess baseline T2DM risk status among participants of the BeWEL trial [a 12‐month randomized controlled trial of a weight loss intervention in people with a body mass index (BMI) > 25 kg/m^2^ and an adenoma detected through the national bowel screening programme] and to explore the implications for diabetes prevention strategies within the CRC screening setting 14.

## Method

All screening participants who had a colorectal adenoma removed in three Scottish health boards (Tayside, Forth Valley and Ayrshire and Arran) between November 2010 and April 2012 (and between January and April 2012 in Greater Glasgow Health Board) were sent a letter of endorsement for participation in the BeWEL study by the lead area CRC clinician and 2 weeks later an invitation letter, brief participant information sheet and reply slip with a prepaid envelope to encourage response.

Those interested in taking part were screened by telephone to assess eligibility (exclusions were BMI < 25 kg/m^2^, insulin‐dependent diabetes and CRC diagnosis, age < 50 or > 74 years). Existing diabetes diagnosis was self‐reported. No information on existing CVD diagnosis or medication was recorded.

Those eligible were invited to provide informed consent and undergo baseline measurements. Participants randomized to the intervention group were seen by a lifestyle counsellor and given a personal energy prescription, body weight scales and a counselling session employing motivational and behavioural techniques for altering diet and physical activity 15. The primary outcome of the trial was weight change over 1 year. The full trial protocol is described elsewhere 16.

The baseline and follow‐up measures included:

1. Body weight – measured with participants wearing indoor clothing and no shoes using a calibrated scale.
2. Height – measured with a mobile stadiometer.
3. Waist circumference – measured with participants in the standing position, either midway between the lateral lower rib margin and the ileac crest or at the level of the umbilicus if these landmarks could not be identified. Two measurements were taken postexhalation and the mean recorded.
4. Blood pressure – taken after the participant had been seated for 5 min with the arm supported at heart level, using an appropriately sized cuff on the left arm and a digital blood pressure monitor. Two readings, or three if noted to be elevated, ≥ 1 min apart, were taken and the mean reported.
5. Fasting plasma glucose (FPG), insulin, glycated haemoglobin (HbA1c) and lipid profiles (cholesterol, low‐density lipoprotein cholesterol, high‐density lipoprotein cholesterol, triglyceride) – assessed by standard techniques in the NHS laboratory. The homeostasis model assessment‐estimated insulin resistance (HOMA‐IR) index was used to assess insulin sensitivity: [insulin (μU/ml) × glucose (mmol/l)]/22.5 17.

In addition, a range of psychosocial variables were collected and these are detailed elsewhere 16.

Diabetes risk was estimated as ‘high’ if HbA1c was between 6.0 and 6.4% or FPG between 5.5 and 6.9 mmol/l and as ‘possible diabetes diagnosis’ (subject to results from repeated measures) when HbA1c ≥ 6.5% or FPG ≥ 7 mmol/l, in accordance with WHO 18 and John *et al*. 19 as utilized by NICE 20.

Data were entered into the statistical package spss (version 19, SPSS, Chicago, Illinois, USA). Descriptive statistics of the cardiometabolic risk factors were used to characterize the cohort in relation to the risk of diabetes. The study obtained ethics approval from the Tayside committee on Medical Ethics B (REC reference number 10/S1402/34) and all participants gave written informed consent before taking part.

## Results

Almost half (49%) of the 997 patients invited to participate expressed an interest in the lifestyle intervention study. Following exclusions for BMI < 25 kg/m^2^ (22%), informed decisions not to participate (9%) and late responses (3%), randomization was carried out on 329 Caucasian participants (243 men and 86 women) with a mean age of 64 years (SD ± 8). Around half of the male participants (51%) and 21% of the women reported never having previously tried to lose weight. Full details of respondents are reported elsewhere 14.

Of the 250 participants assessed for risk of T2DM, 122 were randomly allocated to the intervention group and 128 to the control group. At 12 months' follow‐up 103 in the intervention group and 109 in the control group attended for follow‐up measures, a slightly lower level of study retention at 12 months (84 and 85% respectively) than the full trial (95 and 91%). Reasons for dropping out from the full trial (*n *=* *24) were health concerns (21%), personal reasons (8%) unable to commit (8%) moving from the area (4%), death (4%) and not comfortable with the study (4%); 50% provided no reason.

A preexisting diagnosis of T2DM was made in 47 (14.3%) of the 329 participants. Of the remainder with complete biochemistry results (*n *=* *250), 19 (7.6%) were classified as having potentially *undiagnosed* T2DM (subject to repeat analysis) and 125 (50.0%) as being at high risk on the basis of HbA1c or FPG levels. There were no differences in sociodemographic character between those assessed at high or low risk of T2DM.

**Figure 1:**
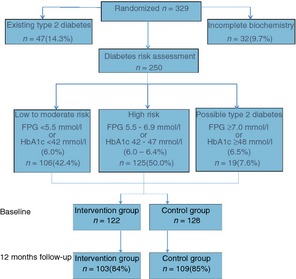
Flowchart identifying the risk of type 2 diabetes among a population of adults screening positive for adenoma. FPG, fasting plasma glucose; HbA1c, glycated haemoglobin.

In keeping with the overweight inclusion criterion, high waist circumference was notable in all categories. More than a quarter of participants in all risk‐assessed categories had raised cholesterol, triglycerides and hypertension indicative of raised risk for CVD (Table 1). Of those assessed at high risk or with potentially undiagnosed T2DM and plasma insulin levels measured to allow HOMA‐IR calculation (*n *=* *113), 81 (71.7%) had HOMA levels > 1.7 molar units which also demonstrates high levels of insulin resistance in this cohort (Fig. 1).

**Table 1:** Cardiovascular risk factors among adults screening positive for adenoma categorized by risk of type 2 diabetes mellitus (T2DM) in four Scottish health boards from November 2010 to January 2012

	Men, mean (SD) (*n *=* *189; 75.6%)	Women, mean (SD) (*n* = 61; 24.4%)	Raised risk definition	Low/moderate T2DM risk^a^ *(n* = 106, 42.4%)	High T2DM risk^a^ (*n *=* *125, 50.0%)	Possible T2DM diagnosis^a^ (*n *=* *19, 7.6%)
BMI (kg/m^2^)	30.3 (3.9)	30.9 (4.7)	Overweight BMI ≥ 25 kg/m^2^	66 (62.3)	67 (53.6)	6 (31.6)
			Obese BMI ≥ 30 kg/m^2^	40 (37.7)	58 (46.4)	13 (68.4)
Waist circumference (cm)	105 (10)	100 (12)	≥ 94 cm men^b^	96 (90.6)	117 (93.6)	18 (94.7)
			≥ 80 cm women^b^			
Plasma triglycerides (mmol/l)	1.7 (1.1)	1.6 (1.0)	≥ 150 mg/dl (1.7 mmol/l)	49 (46.2)	77 (61.6)	15 (78.9)
Total Cholesterol (mmol/l)	5.2 (1.2)	5.6 (1.0)	≥ 6.0 mmol/l	33 (31.1)	36 (28.8)	8 (42.1)
Plasma HDL‐C (mmol/l)	1.34 (0.40)	1.51 (0.33)	Men < 40 mg/dl(1.03 mmol/l)	15 (14.2)	22 (17.6)	6 (31.6)
			Women < 50 mg/dl (1.29 mmol/l)			
Plasma LDL‐C (mmol/l)^c^	3.08 (1.04)	3.41 (0.99)	≥ 2.6 mmol/l	73 (68.9)	80 (66.1)	10 (58.8)
Systolic BP (mmHg)	143 (18)	140 (14)	≥ 130 mmHg	71 (67.0)	101 (80.8)	17 (89.5)
Diastolic BP (mmHg)	86 (10)	80 (8)	≥ 85 mmHg	45 (42.5)	69 (55.2)	13 (68.4)
			Both systolic ≥ 130 mmHg and diastolic ≥ 85 mmHg	44 (41.5)	63 (50.4)	13 (68.4)
HOMA‐IR^d^	2.8 (2.5)	2.6 (2.6)	> 1.7	26 (34.7)	70 (70.0)	11 (84.6)

BMI, body mass index; HDL‐C, high‐density lipoprotein cholesterol; LDL‐C, low‐density lipoprotein cholesterol; BP, blood pressure; HOMA‐IR, homeostasis model assessment‐estimated insulin resistance index.

a *n* (%) Valid percentages are reported.

b International Diabetes Federation definition of central obesity for Europid men and women.

c Plasma LDL‐C was only calculated if plasma triglycerides were < 4.0 mmol/l (Tayside and Forth Valley) or < 4.4 mmol/l (Ayrshire and Arran) (*n *=* *244).

d *n *=* *188.

Reanalysis of T2DM risk at 12 months showed that 21 (20.4%) of the intervention participants improved their risk category compared with 11 (10.2%) in the control group [OR 2.26 (95% CI 1.03–4.96); *P *=* *0.04].

## Discussion

The present study highlights a population group with a high rate of T2DM and many more with metabolic markers indicative of a high risk of diabetes and CVD who have accepted invitations for CRC screening and completed appropriate NHS treatments to reduce the risk of cancer. It is likely that some of those with incomplete biochemical results may also have had indications of raised diabetes risk.

People who are overweight are more likely to develop type 2 diabetes, and the risk rises with increasing body weight 21. Evidence suggests that a 1 kg/m^2^ increase in BMI increases the risk of developing new‐onset type 2 diabetes by 8.4% 22. Lifestyle programmes are the cornerstone of diabetes prevention and have additional cardiovascular benefits with considerable potential to delay future morbidity and associated health‐care costs.

Health‐care settings which include a large number of overweight, older patients provide plausible opportunities for T2DM risk assessment. Whilst it is recognized that people with health conditions such as CVC and polycystic ovary syndrome may be at increased risk of diabetes, little attention has been given to people with colorectal adenomas despite consistent epidemiological evidence of an association between the two conditions. A recent systematic review and meta‐analysis reported that the overall HR for CRC incidence was 1.26 in people with diabetes compared with those without 11. In addition, mortality due to cancer is increased among patients with diabetes, although it is thought that some drug therapies used in diabetes management may modify cancer risk 23. Little is known about the relationship between diabetes and high‐risk adenomas, and while the current study is underpowered to investigate this area future work could add evidence to support lifestyle change in this patient group.

Our previous qualitative work has highlighted the absence of advice on diet, physical activity and body weight in this population group and the importance of clinician endorsement for lifestyle change 24.The colonoscopy setting presents an underexplored opportunity or ‘teachable moment’ to identify and support patients at high risk of cardiometabolic disorders. It is also likely that the potential to reduce cancer risk is a major incentive to engage in lifestyle programmes 25. It is estimated that around 3500 people in Scotland are diagnosed with colorectal adenomas every year 26, most of whom are aged over 50; in line with the general population and the current study, 80% of men and 70% of women have excess body weight. As part of routine procedures prior to colonoscopy, height, weight and blood pressure are usually measured thus allowing the capture of four of the seven key factors for diabetic risk screening (age, gender, hypertension, BMI). The addition of ethnicity, family history of diabetes and the measurement of waist circumference could provide a rapid diabetes screening tool as recommended by NICE 20.

A further reason for utilizing this setting for preventative action comes from the results of the BeWEL intervention trial which demonstrated that people with increased T2DM risk who have had a cancer scare (including men who may not have previously attempted to lose weight) are interested in lifestyle change and are able to lose and maintain clinically relevant amounts of body weight.

However, it is important to consider how successful weight management interventions might be delivered in routine health care given the paucity of NHS staff trained in giving advice on lifestyle change. These are similar issues to those of smoking cessation, which have been addressed by increasing access to a NHS smoking cessation counsellor (in addition to offering community group support, etc.). This approach also deserves consideration with respect to weight management.

These results suggest a diagnosis of adenoma in overweight patients deserves serious consideration for opportunistic screening for diabetes risk with additional opportunities for CVD appraisal. The BeWEL trial also suggests that this setting provides an opportunity for delivering lifestyle interventions in patients who may not previously have attempted to initiate weight loss (notably in men) with a strong likelihood of achieving clinically relevant results for reduction in multiple morbidities including reduced cancer risk.

## Funding

Financial support was provided by the National Prevention Research Initiative (http://www.mrc.ac.uk/research/initiatives/national-prevention-research-initiative-npri/), grant award number G0802030. The National Prevention Research Initiative is a national research initiative administered by the Medical Research Council made up of the following funding partners: Alzheimer's Research Trust; Alzheimer's Society; Biotechnology and Biological Research Council; Cancer Research UK; Chief Scientist Office; Scottish Government Health Directorate; Department of Health; Diabetes UK; Economic and Social Research Council; Engineering and Physical Sciences Research Council; Health & Social Care Research and Development Office for Northern Ireland; Medical Research Council; Welsh Assembly Government and WCRF. Further financial support was provided by NHS Research Scotland to carry out this work.

The Health Services Research Unit, University of Aberdeen, is core funded by the Chief Scientist Office of the Scottish Government Health Directorates.

## Author contributions

RJCS and ASA (guarantors) had the original idea for the study and, with AMC, SC, JJFB, ST and RJS, carried out the design. ASA and RJS obtained funding. AMC and SC were responsible for data collection. MM carried out the analysis. ASA and MM drafted the manuscript which was revised by all authors. All researchers were independent from funders.

The study sponsor and funder played no role in study design, the collection, analysis and interpretation of data, the writing of the report or in the decision to submit the article for publication.

All authors, external and internal, had full access to all of the data (including statistical reports and tables) in the study and can take responsibility for the integrity of the data and the accuracy of the data analysis.

## Ethical approval

Ethics committee approval was granted by Tayside Committee on Medical Research Ethics B on 16 July 2010 (REC ref no 10/S1402/34).

## Data sharing

No additional data available.
